# Perceived academic demands and psychological distress among Chinese university students: structural associations involving chronic time pressure and leisure-time physical activity

**DOI:** 10.3389/fpubh.2026.1984531

**Published:** 2026-10-05

**Authors:** Zhikai Gong, Gang Shen, Zhuang Zhou

**Affiliations:** School of Physical Education, Changzhou University, Changzhou, Jiangsu, China

**Keywords:** chronic time pressure, leisure-time physical activity, perceived academic demands, psychological distress, structural equation modeling, university students

## Abstract

**Objectives:**

This study examined the direct and specific indirect associations among perceived academic demands, chronic time pressure, leisure-time physical activity, and psychological distress in Chinese university students.

**Methods:**

A cross-sectional online survey included 1,390 undergraduates from 10 higher-education institutions in Jiangsu Province, China. Perceived academic demands, chronic time pressure, and psychological distress were modeled as latent variables, whereas log-transformed leisure-time physical activity was modeled as an observed variable. Confirmatory factor analysis and covariate-adjusted structural equation modeling were conducted. Specific indirect associations were estimated using 5,000 bootstrap resamples with bias-corrected 95% confidence intervals.

**Results:**

The adjusted structural model showed good fit (CFI = 0.966, TLI = 0.959, RMSEA = 0.034, SRMR = 0.024). Perceived academic demands were positively associated with chronic time pressure (β = 0.539) and psychological distress (β = 0.382). Chronic time pressure was positively associated with psychological distress (β = 0.334) and negatively associated with leisure-time physical activity (β = −0.179), whereas leisure-time physical activity was negatively associated with distress (β = −0.097; all *p* < 0.001). The specific indirect association through chronic time pressure was *B* = 0.182 (95% bias-corrected CI [0.134, 0.236]), whereas the serial specific indirect association additionally involving leisure-time physical activity was considerably smaller (*B* = 0.010, 95% bias-corrected CI [0.005, 0.018]).

**Conclusions:**

The indirect association through chronic time pressure was substantially larger than the serial indirect association additionally involving leisure-time physical activity, indicating that leisure-time physical activity made only a minimal additional contribution within the concurrent structural model. These findings highlight chronic time pressure as a relevant correlate of student psychological distress and support further investigation of academic time organization alongside opportunities for physical activity. Because the data were cross-sectional, temporal mediation and causal direction cannot be established.

## 1 Introduction

University students experience a substantial burden of mental-health problems during a period when many common mental disorders first emerge or become clinically salient. The World Mental Health International College Student Initiative documented a high prevalence of mental disorders across countries (1), and a recent umbrella review similarly identified substantial burdens of depression, anxiety, sleep problems, and stress-related outcomes in university populations (2). In China, a 2025 systematic review and meta-analysis also reported a considerable prevalence of depressive symptoms among university students (3). These problems are relevant not only to individual well being but also to students' academic functioning and continuation in higher education. Financial, health, romantic, family, and work/school relationship stressors have been associated with common mental disorders (4), while depression and stress are associated with a greater likelihood of discontinuing higher education (5).

University mental-health interventions have largely focused on individual-level approaches. Review-level evidence includes cognitive-behavioral, mindfulness, psychoeducational, relaxation, and related psychological interventions, whereas evidence for setting-based approaches remains more limited (6). Research among Chinese undergraduates has similarly emphasized social support and individual coping strategies (7). These approaches remain important, but they do not fully address whether features of academic life contribute to psychological distress. Meta-analytic evidence linking student stress with poorer emotional well being supports closer attention to the conditions under which stress arises and persists (8). Examining academic demands, students' perceptions of available time, and health-related behaviors may therefore provide a broader account of psychological distress in university settings.

The Study Demands-Resources (SD-R) framework provides a useful basis for examining academic demands as characteristics of the study environment rather than as psychological outcomes themselves (9, 10). Conservation of Resources Theory complements this perspective by emphasizing the strain that may arise when valued resources such as time, energy, and perceived control are repeatedly consumed or threatened (11). This distinction is important because academic stress is often defined broadly to include workload, examinations, performance expectations, time constraints, self-appraisal, and emotional responses (12–14). In the present study, perceived academic demands were defined more narrowly as students' perceptions of workload, required effort, sustained attention, and information-processing demands. Chronic time pressure was treated separately as the persistent subjective experience that available time is insufficient.

Empirical evidence supports the relevance of academic demands to student well being. The Study Demands and Resources Scale has shown longitudinal measurement stability and criterion validity in university populations (15), and recent research has linked greater academic demands with greater emotional exhaustion (16). Longitudinal studies also suggest that academic and time-management conditions may extend beyond emotional outcomes: greater non-physical-education course load has been associated with poorer physical-fitness outcomes (17), while time-management pressure has been linked with exercise habits and physical fitness among Chinese undergraduates (18). Chronic time pressure captures a related but distinct experience. The Chronic Time Pressure Inventory was developed to assess persistent perceptions of insufficient time and feeling rushed (19), and subsequent psychometric studies broadly support an overall time-pressure construct (20, 21). Students with similar formal workloads may therefore differ in the extent to which they experience their available time as sufficient.

Research on time poverty further supports the relevance of subjective time scarcity. Time is a finite resource that must be distributed across study, recovery, relationships, and health-related activities (22). Perceived time pressure has been associated with depressive symptoms and has been examined as a mechanism linking social roles with depression (23), and a three-wave study of university students found that time pressure predicted subsequent exhaustion, which in turn predicted health-related productivity loss (24). On this basis, we hypothesized that perceived academic demands would be positively associated with chronic time pressure (H1) and psychological distress (H3), and that chronic time pressure would be positively associated with psychological distress (H4). We also expected a positive specific indirect association between perceived academic demands and psychological distress through chronic time pressure (H6).

Chronic time pressure may also influence how students allocate time to health-related behaviors. From a resource perspective, time is finite, and greater perceived scarcity may increase competition among study, recovery, social activities, and health behaviors (11, 22). Experimental evidence provides support for this possibility. In a 4-week mixed experimental study of university students, higher goal conflict and time pressure were associated with lower health-behavior motivation, with the largest decline in implementation attitudes occurring when both conditions were high (25). Behavioral intentions were more stable, suggesting that time pressure may affect the motivational and practical conditions surrounding health behavior rather than uniformly reducing behavior itself. Longitudinal evidence also indicates that academic and time-management conditions may be relevant to students' physical-fitness outcomes and exercise patterns: greater non-physical-education course load was associated with poorer physical-fitness outcomes over 3 years (17), while a 4-year Chinese study identified time-management pressure as a negative factor within a broader model of exercise habits and physical fitness (18).

Physical activity appears particularly sensitive to competing demands on students‘ time. Systematic reviews have identified lack of time, academic commitments, competing responsibilities, environmental opportunity, behavioral regulation, and motivational factors as important influences on physical activity among university students (26, 27). Qualitative research provides a more detailed account of these constraints. Students have reported that sport or exercise may be among the first activities reduced or canceled during periods of time pressure, while academic schedules, convenience, social context, and competing responsibilities jointly shape participation (28, 29). Evidence from Chinese university students is consistent with these findings. A recent COM-B/TDF-based qualitative study identified academic pressure and time-management difficulties as barriers to physical activity, whereas making use of fragmented time and integrating activity into everyday routines were described as facilitators (30). These findings make perceived time pressure a relevant factor to examine in relation to students' opportunities for physical activity.

The present study therefore focused specifically on leisure-time physical activity. Unlike physical activity undertaken as part of work or study obligations, active transport, or institutionally scheduled physical education, leisure-time activity is more discretionary and may be particularly sensitive to whether students perceive that they have sufficient time for exercise or recreation. At the same time, leisure-time physical activity is influenced by many factors beyond academic demands or time pressure, including habit, competence, social support, access to facilities, environmental opportunity, and motivation (27, 30). Greater academic demands should therefore not be assumed to correspond directly to lower leisure-time activity. Instead, the more specific expectation was that chronic time pressure would be negatively associated with leisure-time physical activity (H2). The direct association between perceived academic demands and leisure-time physical activity was examined as an exploratory path.

Leisure-time physical activity was also included because of its relevance to student mental health. Intervention evidence in university populations generally suggests mental-health benefits, although findings vary across outcomes and study designs. A systematic review and meta-analysis of undergraduate interventions reported reductions in anxiety, depression, and stress following physical-activity interventions, while also noting substantial heterogeneity and limitations in the certainty of evidence (31). A separate systematic review of physical-activity interventions in higher-education students similarly found improvements in mental-health or quality-of-life outcomes across a substantial proportion of included studies (32). A meta-analysis focusing specifically on depression among college students also reported beneficial effects of physical exercise (33). This intervention literature provides a rationale for including physical activity as a behavioral correlate of student psychological health.

Prospective and longitudinal evidence further supports an association between physical activity and mental health while also indicating variation across activity intensities and over time. Meta-analyses of prospective cohort studies across broader populations have associated greater physical activity with a lower subsequent risk of depression (34, 35). More directly relevant to university students, a 2026 meta-analysis of 22 studies involving 66,683 participants found lower odds of depressive symptoms among students with higher physical activity (36). University-specific longitudinal studies, however, indicate that the pattern may differ by activity intensity and over time. A four-wave study found that vigorous leisure-time physical activity was inversely associated with depressive symptoms at baseline, but changes in moderate or vigorous activity were not associated with changes in depressive symptoms (37). Another longitudinal study found no association between changes in vigorous or moderate leisure-time activity and depressive-symptom trajectories, whereas greater light activity was associated with slower symptom growth (38).

Evidence from Chinese university students is broadly consistent with this pattern. A large study across six geographic regions reported inverse associations between moderate-to-vigorous physical activity and psychological symptoms among university students (39), while a health survey involving more than 40,000 Chinese university students linked greater moderate-to-vigorous physical activity with lower levels of depression (40). Other university studies have associated lower exercise participation with poorer mental health and suicidality (41), and vigorous physical activity with lower perceived stress, with socializing partly mediating associations with mental health and stress (42). On the basis of this evidence, we hypothesized that greater leisure-time physical activity would be negatively associated with psychological distress (H5).

Although previous studies have examined academic demands, time pressure, physical activity, and student mental health, these relationships have largely been studied separately. Research on academic demands has mainly focused on stress, exhaustion, and well being, whereas studies of time pressure have emphasized subjective time scarcity and competing demands. Physical-activity research, in turn, has typically examined barriers to participation or associations with mental-health outcomes. Less is known about how these factors are related when considered simultaneously. The present study therefore examined perceived academic demands, chronic time pressure, leisure-time physical activity, and psychological distress within a single structural model. This approach allowed us to assess whether chronic time pressure was associated with both psychological distress and leisure-time physical activity, while also estimating the association between leisure-time physical activity and psychological distress.

The serial specific indirect association provided a further test of whether leisure-time physical activity was additionally involved in the association through chronic time pressure. If greater perceived academic demands are associated with greater chronic time pressure (H1), greater chronic time pressure with lower leisure-time physical activity (H2), and greater leisure-time physical activity with lower psychological distress (H5), these paths together imply a positive serial indirect association between perceived academic demands and psychological distress through chronic time pressure and leisure-time physical activity. Taken together, these lines of evidence suggest that chronic time pressure may be accompanied not only by greater psychological distress but also by lower engagement in discretionary health behavior, providing a basis for examining leisure-time physical activity within the same structural sequence. We therefore hypothesized this serial indirect association (H7) and compared its magnitude with the indirect association through chronic time pressure alone (H6). The direct path from perceived academic demands to leisure-time physical activity remained exploratory. Because the focal variables were measured at the same time point, the indirect associations were examined as concurrent structural relationships. Figure 1 presents the hypothesized model.

**Figure 1:**
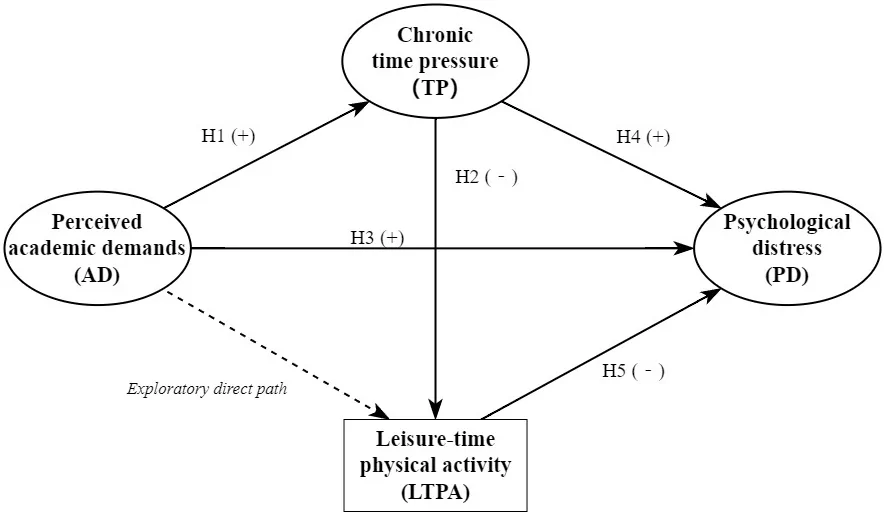
Hypothesized structural model. AD, perceived academic demands; TP, chronic time pressure; LTPA, leisure-time physical activity; PD, psychological distress. AD, TP, and PD were modeled as latent constructs, whereas LTPA was modeled as an observed variable. Solid arrows represent H1–H5; the dashed AD-to-LTPA path was exploratory. H6 specifies the specific indirect association AD → TP → PD, and H7 specifies the serial specific indirect association AD → TP → LTPA → PD.

## 2 Materials and methods

### 2.1 Study design and context

A cross-sectional online survey was conducted among full-time undergraduate students from 10 higher-education institutions in Jiangsu Province, China, including institutions located in Changzhou, Zhenjiang, Suzhou, and Wuxi. The study used multi-site, non-probability convenience sampling, with participating institutions and recruitment channels selected on the basis of accessibility and institutional cooperation. The sample should therefore not be interpreted as a probability sample of university students in Jiangsu Province. Data were collected from May 30 to June 15, 2026. The same online questionnaire was administered across participating institutions through Wenjuanxing, a widely used web-based survey platform in China, and distributed through established student communication channels. Participation was voluntary, and students reviewed the study information and provided electronic informed consent before completing the questionnaire. To reduce duplicate submissions, the platform was configured to permit one submission per registered account. Reporting was informed by the STROBE statement for cross-sectional studies and relevant CHERRIES items for web-based surveys (43, 44).

### 2.2 Participants and data quality screening

Eligible participants were full-time undergraduate students aged 18–25 years who were able to complete the questionnaire in Chinese and provided electronic informed consent. Before data collection, the planned recruitment target was approximately 1,400 undergraduates across the 10 participating institutions. This was a pragmatic multi-site recruitment target rather than a model-specific a priori power-based minimum; no formal a priori SEM power calculation was performed. A total of 1,451 questionnaire records were initially available. During preliminary data cleaning, 45 records were excluded because of clear response-quality concerns: 20 were exact duplicates of earlier questionnaire records across the retained questionnaire fields, and 25 showed highly patterned responding across the multi-item scales. This left 1,406 complete questionnaires for formal response-quality screening before model estimation. Response quality was then evaluated using two operational indicators: completion time shorter than 60 s and a longest run of at least six identical consecutive responses. A questionnaire was excluded only when both indicators were present. Sixteen questionnaires met this conjunctive criterion, leaving 1,390 participants in the final analytic sample. Because only complete questionnaires entered the analytic dataset, no missing-data imputation was required. No observations were excluded because of extreme values or combinations of the focal variables, or because they adversely affected model fit. High leisure-time physical-activity values were retained when the reported frequency and duration remained logically permissible.

### 2.3 Measures

All study measures were administered in Chinese. Perceived academic demands, chronic time pressure, and psychological distress were modeled as latent variables using item-level indicators, whereas leisure-time physical activity was modeled as an observed behavioral variable. Measures without an established Chinese version were translated into Chinese and reviewed by bilingual researchers for semantic and conceptual equivalence before administration.

#### 2.3.1 Perceived academic demands

Perceived academic demands were assessed using the five-item workload dimension of the Study Demands and Resources Scale (15). For the present analysis, four items (AD1, AD3, AD4, and AD5) were retained to represent perceived academic demands. These items assessed the amount of study work, the effort required to complete academic tasks, sustained attention, and information-processing demands. AD2, which explicitly referred to studying or completing tasks under time pressure, was administered but excluded from the analyzed indicator set to reduce direct content overlap with chronic time pressure. Participants responded with reference to the previous 4 weeks on a five-point scale from 1 (never) to 5 (always), with higher scores indicating greater perceived academic demands. The four retained items were modeled as indicators of a latent factor. Cronbach's α was 0.835.

#### 2.3.2 Chronic time pressure

Chronic time pressure was assessed using the 13-item Chronic Time Pressure Inventory (CTPI) (19), which captures persistent perceptions of insufficient time and feeling rushed. Participants rated their experiences during the previous 4 weeks on a five-point scale from 1 (strongly disagree) to 5 (strongly agree). TP2, TP6, and TP9 were reverse scored, and higher scores indicated greater chronic time pressure. All 13 items were retained as indicators of a single latent factor. Previous Rasch and CFA/ESEM evaluations have generally supported the overall construct while identifying some variability in item performance (20, 21); therefore, item loadings and overall measurement quality were evaluated explicitly rather than deleting items solely on the basis of lower loadings in the present sample. Cronbach's α was 0.930.

#### 2.3.3 Leisure-time physical activity

Physical activity was assessed using items from the World Health Organization Global Physical Activity Questionnaire (GPAQ) (45, 46). Items covering work- or study-related physical activity, walking or cycling for transport, leisure-time physical activity, and sedentary time were administered. The present analysis focused specifically on leisure-time physical activity because it represents a comparatively discretionary form of activity that may be more sensitive to perceived time constraints. Participants reported the weekly frequency and daily duration of vigorous- and moderate-intensity leisure-time activity. Weekly leisure-time physical activity was calculated using the standard GPAQ MET values of 8 METs for vigorous activity and 4 METs for moderate activity: leisure-time MET-min/week = (8 × vigorous min/week) + (4 × moderate min/week). Work- or study-related physical activity and transport activity were not included in the focal LTPA variable. The sedentary-time item was used only for descriptive reporting. The GPAQ has shown moderate-to-substantial reliability and moderate-to-strong concurrent validity across multiple countries, although criterion validity against objective measures was poor to fair; it has also undergone psychometric evaluation in Chinese university students (45, 47). Leisure-time MET-min/week was markedly right-skewed and included a substantial number of zero values. Raw min and MET-min/week were retained for descriptive reporting, whereas correlations and structural modeling used ln (leisure-time MET-min/week + 1). This transformation preserved participants reporting no leisure-time activity while reducing the influence of the long right tail. The transformed value was modeled as an observed variable.

#### 2.3.4 Psychological distress

Psychological distress was assessed using the six-item Kessler Psychological Distress Scale (K6) (48). Participants reported how often during the previous 30 days they had experienced nervousness, hopelessness, restlessness, depressed mood that could not be cheered up, the feeling that everything was an effort, and worthlessness. Items were rated from 0 to 4 and summed to produce a total score from 0 to 24, with higher scores indicating greater nonspecific psychological distress. The K6 has demonstrated satisfactory psychometric properties among Chinese undergraduates (49). The summed score was used descriptively, whereas the six items were modeled as indicators of a latent psychological-distress factor in CFA and SEM. A total score ≥13 was used only as a descriptive screening indicator and not as a psychiatric diagnosis or criterion for model inclusion. Cronbach's α was 0.903.

### 2.4 Covariates

Covariates were selected on conceptual and methodological grounds to account for non-focal demographic, anthropometric, activity-related, and socioeconomic characteristics that could plausibly be associated with one or more endogenous variables. Age and gender were included as basic demographic characteristics; BMI as an anthropometric characteristic relevant to physical-activity patterns; weekly h of physical education or other university-organized physical activity to account for structured activity exposure distinct from discretionary leisure-time physical activity; and family financial pressure as a non-academic socioeconomic stressor relevant to psychological distress (4, 27).

The adjusted structural model included age, gender, body mass index (BMI), weekly h of physical education or other university-organized physical activity, and family financial pressure as covariates. BMI was calculated from self-reported height and weight. Weekly organized physical activity was reported for the current semester, and family financial pressure was rated on a five-point scale from 1 (none at all) to 5 (very high). Age, BMI, weekly organized physical activity, and family financial pressure were entered as continuous variables. Gender was represented by three dummy variables, with men as the reference category: women, another gender identity, and prefer not to answer. All covariates were specified as predictors of chronic time pressure, leisure-time physical activity, and psychological distress. Perceived academic demands was treated as the focal exogenous predictor and was allowed to covary with the covariates, which were also allowed to covary with one another. Weekly formal class h and extracurricular study h were examined in supplementary correlation analyses with perceived academic demands and chronic time pressure. Daily sleep duration and sitting time were used for descriptive reporting only.

### 2.5 Statistical analysis

Statistical analyses were conducted using IBM SPSS Statistics 26.0 and IBM SPSS Amos 26.0. All tests were two-sided with α = 0.05. Continuous variables were summarized using means and standard deviations or, for markedly skewed distributions, medians and interquartile ranges. Categorical variables were summarized using frequencies and percentages. Scale means were used to describe perceived academic demands and chronic time pressure, and psychological distress was summarized using the K6 total score. Leisure-time physical activity was expressed as MET-min/week for descriptive reporting and transformed as ln (MET-min/week + 1) for correlation and structural analyses. Pearson correlations were used to examine unadjusted associations among the four focal variables.

A three-factor confirmatory factor analysis (CFA) was conducted for perceived academic demands, chronic time pressure, and psychological distress using item-level indicators. Leisure-time physical activity was not included in the CFA because it was modeled as an observed variable. Model fit was evaluated using χ^2^, χ^2^/df, the comparative fit index (CFI), Tucker–Lewis index (TLI), root mean square error of approximation (RMSEA) with 90% confidence intervals, and standardized root mean square residual (SRMR); SRMR was obtained using the Amos Standardized RMR plugin. Model fit was considered across the indices jointly (50). Measurement quality was evaluated using standardized factor loadings, Cronbach's α, composite reliability (CR), average variance extracted (AVE), and the heterotrait-monotrait ratio (HTMT) (51, 52). Construct distinctiveness was further examined by comparing the hypothesized three-factor model with a two-factor model combining perceived academic demands and chronic time pressure and a one-factor model combining all items from the three latent constructs. As a supplementary diagnostic for potential common method variance, Harman's single-factor test was conducted using the 23 retained questionnaire indicators of perceived academic demands, chronic time pressure, and psychological distress. All indicators were entered simultaneously into an unrotated principal-component analysis, and the proportion of total variance explained by the first component was examined (53).

The structural model included paths from perceived academic demands to chronic time pressure and psychological distress, from chronic time pressure to leisure-time physical activity and psychological distress, and from leisure-time physical activity to psychological distress. A direct path from perceived academic demands to leisure-time physical activity was included as exploratory. Parameters were estimated using maximum likelihood. The adjusted model included age, gender, BMI, weekly h of physical education or other university-organized physical activity, and family financial pressure as covariates. Unstandardized coefficients (B), standard errors (SE), standardized coefficients (β), *p* values, and explained variance (*R*^2^) were reported.

Two specific indirect associations were estimated: perceived academic demands → chronic time pressure → psychological distress (H6) and perceived academic demands → chronic time pressure → leisure-time physical activity → psychological distress (H7). Bias-corrected 95% confidence intervals for the unstandardized indirect estimates were obtained from 5,000 bootstrap resamples (54). Standardized specific indirect estimates were calculated as the products of the standardized coefficients along the corresponding paths. Interpretation considered the magnitude of the indirect estimates in addition to their confidence intervals (55). Because all focal variables were measured concurrently, the indirect estimates were interpreted as concurrent structural associations rather than evidence of temporal mediation (56).

Two supplementary analyses were conducted. First, Pearson correlations examined weekly formal class h and extracurricular study h in relation to perceived academic demands and chronic time pressure using the corresponding scale means. Second, the three-factor CFA and adjusted structural model were re-estimated after excluding TP2R, the CTPI item with the lowest standardized loading in the primary CFA. H6 and H7 were also re-estimated using the same 5,000-resample bootstrap procedure.

### 2.6 Ethics

The study protocol was reviewed and approved by the Biomedical Ethics Committee of Changzhou University (approval number 202605210001). Before completing the questionnaire, participants reviewed the online study information and provided electronic informed consent. Participation was voluntary, and the analytic dataset contained no direct personal identifiers. Participants could discontinue the questionnaire at any time before submission.

## 3 Results

### 3.1 Participant flow and characteristics

A total of 1,451 questionnaire records were initially available. During preliminary data cleaning, 45 records were excluded because of response-quality concerns, including 20 exact duplicate questionnaire records and 25 records showing highly patterned responding across the multi-item scales. The remaining 1,406 complete questionnaires underwent formal response-quality screening. Sixteen questionnaires met both response-quality criteria—completion time < 60 s and a longest run of at least six identical consecutive responses—and were excluded, leaving 1,390 participants in the final analytic sample.

Participants had a mean age of 20.12 years (SD = 1.27) and a mean BMI of 21.78 kg/m^2^ (SD = 2.53). The sample included 683 men (49.1%), 660 women (47.5%), 20 participants reporting another gender identity (1.4%), and 27 who preferred not to answer (1.9%). Participants reported a mean of 1.93 h/week (SD = 1.24) of physical education or other university-organized physical activity. Mean family financial pressure was 2.98 (SD = 1.14) on the five-point scale. Grade distribution and additional sample characteristics are presented in Table 1.

**Table 1:** Participant characteristics (*N* = 1,390).

Variable	Result
Age, years	20.12 ± 1.27
BMI, kg/m^2^	21.78 ± 2.53
Men	683 (49.1%)
Women	660 (47.5%)
Other gender	20 (1.4%)
Prefer not to answer	27 (1.9%)
First year	383 (27.6%)
Second year	355 (25.5%)
Third year	356 (25.6%)
Fourth year	260 (18.7%)
Fifth year	36 (2.6%)
Physical education or other university-organized physical activity, h/week	1.93 ± 1.24
Family financial pressure (1–5)	2.98 ± 1.14
Formal class hours, h/week	22.43 ± 5.15
Extracurricular study, h/week	14.00 ± 7.25
Sleep duration, h/day	6.97 ± 0.69
Sitting time, min/day	478.86 ± 114.56
No leisure-time physical activity	429 (30.9%)
K6 total score ≥ 13	208 (15.0%)

Continuous variables are presented as mean ± SD. K6 ≥ 13 is a screening descriptor and does not indicate a clinical diagnosis.

### 3.2 Descriptive statistics and correlations

Mean perceived academic demands were 3.17 (SD = 0.95), mean chronic time pressure was 3.09 (SD = 0.87), and mean K6 psychological distress was 6.65 (SD = 5.52). Median leisure-time physical activity was 70 min/week (IQR 0–144), equivalent to 336 MET-min/week (IQR 0–792), and 429 participants (30.9%) reported no leisure-time physical activity. Perceived academic demands were positively correlated with chronic time pressure (*r* = 0.477) and psychological distress (*r* = 0.501), and chronic time pressure was positively correlated with psychological distress (*r* = 0.520). Leisure-time physical activity was negatively correlated with perceived academic demands (*r* = −0.146), chronic time pressure (*r* = −0.207), and psychological distress (*r* = −0.215). All correlations among the four focal variables were statistically significant at p < 0.001 (Table 2). Weekly formal class h were not significantly correlated with perceived academic demands (*r* = −0.012, *p* = 0.646) or chronic time pressure (*r* = −0.011, *p* = 0.693). Extracurricular study hours were positively correlated with perceived academic demands (*r* = 0.556, *p* < 0.001) and chronic time pressure (*r* = 0.388, *p* < 0.001) (Supplementary Table S4).

**Table 2:** Descriptive statistics, reliability, and correlations among core variables.

Variable	M ±SD or Median (IQR)	α	1	2	3	4
1. Perceived academic demands	3.17 ± 0.95	0.835	1			
2. Chronic time pressure	3.09 ± 0.87	0.930	0.477^***^	1		
3. Leisure-time physical activity	336 (0–792)	—	−0.146^***^	−0.207^***^	1	
4. Psychological distress	6.65 ± 5.52	0.903	0.501^***^	0.520^***^	−0.215^***^	1

Leisure-time physical activity is described in MET-min/week; correlations use ln(MET-min/week + 1). ^***^p < 0.001.

### 3.3 Measurement model

The hypothesized three-factor measurement model showed good fit: χ^2^(227) = 816.39, χ^2^/df = 3.60, CFI = 0.967, TLI = 0.964, RMSEA = 0.043, 90% CI [0.040, 0.046], and SRMR = 0.028. Fit was poorer for the two-factor model combining perceived academic demands and chronic time pressure, χ^2^(229) = 2298.90, CFI = 0.886, TLI = 0.874, and RMSEA = 0.081, and poorest for the one-factor model, χ^2^(230) = 5108.25, CFI = 0.731, TLI = 0.704, and RMSEA = 0.124. These results supported the empirical distinction among perceived academic demands, chronic time pressure, and psychological distress (Table 3). As a supplementary diagnostic of potential common method variance, Harman's single-factor test showed that the first unrotated component accounted for 42.70% of the total variance across the 23 retained questionnaire indicators.

**Table 3:** Comparison of measurement models.

Model	*χ^2^*	df	CFI	TLI	RMSEA
Three-factor model	816.39	227	0.967	0.964	0.043
Two-factor model (perceived academic demands + chronic time pressure)	2298.90	229	0.886	0.874	0.081
One-factor model	5108.25	230	0.731	0.704	0.124

For the three-factor model, RMSEA 90% CI [0.040, 0.046] and SRMR = 0.028. SRMR was not available for the competing models because those models were not re-estimated with the same Amos plugin workflow.

Cronbach's α values were 0.835 for perceived academic demands, 0.930 for chronic time pressure, and 0.903 for psychological distress. Corresponding CR values were 0.835, 0.932, and 0.903, and AVE values were 0.559, 0.519, and 0.609, respectively. HTMT values ranged from 0.537 to 0.571. Standardized factor loadings ranged from 0.736 to 0.762 for perceived academic demands, from 0.394 to 0.816 for chronic time pressure, and from 0.749 to 0.796 for psychological distress (Table 4).

**Table 4:** Reliability, convergent validity, and discriminant validity of the latent constructs.

Construct	Items	Standardized loading range	CR	AVE
Perceived academic demands	4	0.736–0.762	0.835	0.559
Chronic time pressure	13	0.394–0.816	0.932	0.519
Psychological distress	6	0.749–0.796	0.903	0.609

HTMT values ranged from 0.537 to 0.571.

Item wording, standardized factor loadings, squared multiple correlations, and source/adaptation information for all latent-variable indicators are provided in Supplementary Table S1. TP2R had the lowest standardized loading (0.394). A sensitivity CFA excluding TP2R produced closely similar fit: χ^2^(206) = 769.50, χ^2^/df = 3.74, CFI = 0.968, TLI = 0.965, RMSEA = 0.044, 90% CI [0.041, 0.048], and SRMR = 0.028 (Supplementary Table S3).

### 3.4 Structural model and hypothesis testing

The covariate-adjusted structural model showed good fit: χ^2^(387) = 1006.51, χ^2^/df = 2.60, CFI = 0.966, TLI = 0.959, RMSEA = 0.034, 90% CI [0.031, 0.037], and SRMR = 0.024. The model explained 29.9% of the variance in chronic time pressure, 6.3% in leisure-time physical activity, and 43.7% in psychological distress.

Perceived academic demands were positively associated with chronic time pressure (B = 0.583, SE = 0.035, β = 0.539, *p* < 0.001) and psychological distress (B = 0.387, SE = 0.034, β = 0.382, *p* < 0.001). Chronic time pressure was negatively associated with leisure-time physical activity (B = −0.565, SE = 0.107, β = −0.179, *p* < 0.001) and positively associated with psychological distress (B = 0.313, SE = 0.029, β = 0.334, *p* < 0.001). Leisure-time physical activity was negatively associated with psychological distress (B = −0.029, SE = 0.007, β = −0.097, *p* < 0.001). These findings were consistent with H1–H5. The exploratory direct path from perceived academic demands to leisure-time physical activity was not statistically significant (B = −0.197, SE = 0.121, β = −0.058, *p* = 0.105). Structural-path estimates are presented in Table 5 and Figure 2, with complete covariate-path estimates provided in Supplementary Table S2.

**Table 5:** Adjusted structural paths and hypothesis assessments.

Path	*B*	SE	β	*p*	Hypothesis assessment
Perceived academic demands → chronic time pressure	0.583	0.035	0.539	< 0.001	Consistent with H1
Chronic time pressure → leisure-time physical activity	−0.565	0.107	−0.179	< 0.001	Consistent with H2
Perceived academic demands → psychological distress	0.387	0.034	0.382	< 0.001	Consistent with H3
Chronic time pressure → psychological distress	0.313	0.029	0.334	< 0.001	Consistent with H4
Leisure-time physical activity → psychological distress	−0.029	0.007	−0.097	< 0.001	Consistent with H5
Perceived academic demands → leisure-time physical activity	−0.197	0.121	−0.058	0.105	Exploratory path; not statistically significant

The model was adjusted for age, gender, BMI, weekly hours of physical education or other university-organized physical activity, and family financial pressure.

**Figure 2:**
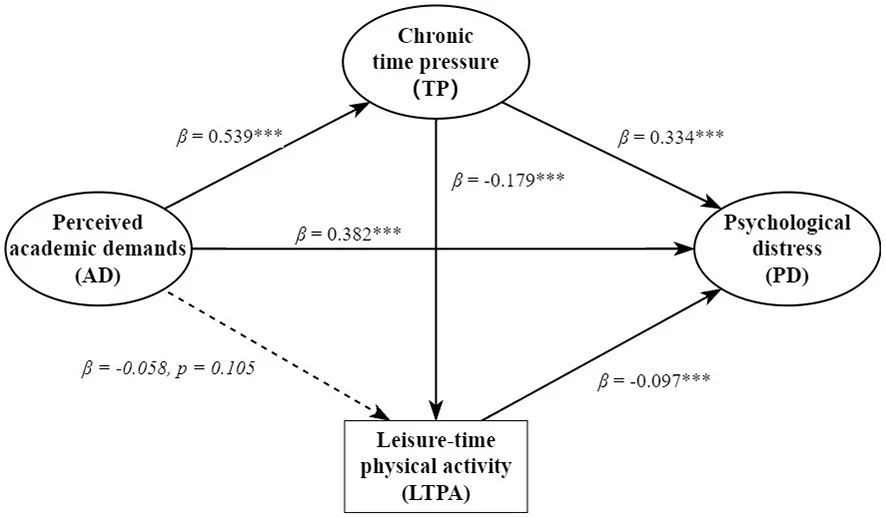
Final adjusted structural model with standardized coefficients. The model adjusted for age, three gender dummy variables (men as the reference category), BMI, weekly h of physical education or other university-organized physical activity, and family financial pressure; covariate paths are omitted from the diagram for clarity. AD, TP, and PD were modeled as latent constructs, whereas LTPA was modeled as an observed variable. Solid arrows indicate statistically significant paths; the dashed arrow indicates the nonsignificant exploratory path. ***p < 0.001.

In the sensitivity model excluding TP2R, model fit remained closely similar: χ^2^(358) = 944.51, CFI = 0.968, TLI = 0.961, RMSEA = 0.034, 90% CI [0.032, 0.037], and SRMR = 0.023. Focal standardized path estimates differed from those in the primary model by no more than 0.002, and the *R*^2^ values for chronic time pressure, leisure-time physical activity, and psychological distress were unchanged (Supplementary Table S3).

### 3.5 Specific indirect associations

Both hypothesized specific indirect associations had bias-corrected bootstrap confidence intervals that excluded zero. The indirect association from perceived academic demands to psychological distress through chronic time pressure was B = 0.182 (bootstrap SE = 0.026, 95% bias-corrected CI [0.134, 0.236]; standardized specific indirect estimate = 0.180), consistent with H6. The serial indirect association through chronic time pressure and leisure-time physical activity was B = 0.010 (bootstrap SE = 0.003, 95% bias-corrected CI [0.005, 0.018]; standardized specific indirect estimate = 0.009). Although the confidence interval excluded zero, the standardized magnitude was very small. The serial estimate was substantially smaller than the indirect association through chronic time pressure alone (Table 6).

**Table 6:** Specific indirect associations in the adjusted model.

Path	*B*	Bootstrap SE	95% BC CI	βind	Hypothesis assessment
Perceived academic demands → chronic time pressure → psychological distress	0.182	0.026	[0.134, 0.236]	0.180	Consistent with H6
Perceived academic demands → chronic time pressure → leisure-time physical activity → psychological distress	0.010	0.003	[0.005, 0.018]	0.009	95% BC CI excluded zero; very small in magnitude

BC CI, bias-corrected bootstrap confidence interval. Bootstrap resamples = 5,000. B and its confidence interval are unstandardized specific indirect estimates. βind is the standardized path-product estimate calculated from the standardized coefficients along the corresponding indirect sequence; bootstrap confidence intervals refer to B.

In the sensitivity model excluding TP2R, the corresponding estimates were nearly unchanged: B = 0.182 (95% bias-corrected CI [0.133, 0.236]) for H6 and B = 0.010 (95% bias-corrected CI [0.005, 0.018]) for H7 (Supplementary Table S3).

## 4 Discussion

### 4.1 Principal findings

Perceived academic demands were positively associated with chronic time pressure (β = 0.539) and psychological distress (β = 0.382). Chronic time pressure was also positively associated with psychological distress (β = 0.334) and negatively associated with leisure-time physical activity (β = −0.179). Leisure-time physical activity was inversely associated with psychological distress, although the association was small (β = −0.097). The exploratory direct association between perceived academic demands and leisure-time physical activity was not statistically significant. The indirect association through chronic time pressure alone (B = 0.182) was substantially larger than the serial indirect association additionally involving leisure-time physical activity (B = 0.010). Thus, leisure-time physical activity made only a minimal additional contribution within the concurrent structural model.

### 4.2 Perceived academic demands, chronic time pressure, and psychological distress

The positive association between perceived academic demands and chronic time pressure is consistent with the Study Demands-Resources framework (9, 10) and Conservation of Resources Theory (11). Academic demands and chronic time pressure were related but empirically distinct: the perceived-academic-demands measure focused on workload, effort, sustained attention, and information-processing requirements, while chronic time pressure reflected students' persistent sense that available time was insufficient. The three-factor measurement model also fit substantially better than the model combining these two constructs.

Previous studies provide a similar pattern of evidence. Academic demands have been associated with emotional exhaustion among university students (16), and longitudinal research has linked time pressure with subsequent exhaustion and related productivity loss (24). Perceived time pressure has also been associated with depressive symptoms and examined as a mechanism linking social roles with depression (23). In the present study, extracurricular study h were positively correlated with both perceived academic demands (*r* = 0.556) and chronic time pressure (*r* = 0.388), whereas formal class h were not significantly correlated with either construct. These findings suggest that students' experience of academic demands and time scarcity may not be captured fully by scheduled class time alone.

Both perceived academic demands and chronic time pressure were positively associated with psychological distress in the adjusted model. The direct association between perceived academic demands and distress remained substantial after chronic time pressure and the other model variables were included, indicating that time pressure represents only part of the broader association. Other processes may also be relevant, including performance concerns, examination and workload pressures, and broader personal or interpersonal stressors (4, 12–14). The indirect association through chronic time pressure should therefore be understood as one component of the concurrent relationship between perceived academic demands and psychological distress.

### 4.3 Chronic time pressure and leisure-time physical activity

Chronic time pressure was negatively associated with leisure-time physical activity. This finding is consistent with previous evidence identifying lack of time and competing responsibilities as common barriers to physical activity among university students. Systematic reviews have highlighted academic commitments, time constraints, environmental opportunity, and behavioral regulation as important influences on activity participation (26, 27). Qualitative studies similarly show that exercise is often reduced when academic and scheduling pressures increase (28, 29). Among Chinese university students, academic pressure and time-management difficulties have also been identified as barriers, whereas using fragmented time and integrating activity into daily routines may facilitate participation (30).

The exploratory direct association between perceived academic demands and leisure-time physical activity was not statistically significant after chronic time pressure was included. Importantly, this non-significant exploratory direct path is separate from H7, which was defined by the sequence perceived academic demands → chronic time pressure → leisure-time physical activity → psychological distress and therefore did not require a direct perceived academic demands → leisure-time physical activity association. This finding suggests that greater academic demands do not necessarily correspond to lower leisure-time activity in a simple or uniform manner. Previous longitudinal studies have linked greater non-physical-education course load with poorer physical-fitness outcomes (17) and time-management pressure with less favorable exercise habits (18), but these measures capture somewhat different aspects of students' academic and temporal conditions. The present results therefore support considering perceived academic demands and subjective time pressure separately when examining discretionary physical activity.

Experimental evidence also suggests that time pressure may influence the motivational conditions surrounding health behavior. In a 4-week mixed experiment, high goal conflict and time pressure were associated with lower health-behavior motivation, with the strongest decline in implementation attitudes under combined high conflict and high pressure (25). In the present study, however, the model explained only 6.3% of the variance in leisure-time physical activity. This relatively modest explanatory power indicates that activity participation was also shaped by factors beyond chronic time pressure, including habit, competence, social support, accessibility, and environmental opportunity (27, 30).

### 4.4 Leisure-time physical activity, psychological distress, and the serial specific indirect association

Leisure-time physical activity was inversely associated with psychological distress after adjustment for perceived academic demands, chronic time pressure, and the covariates, although the standardized association was small (β = −0.097). This finding is broadly consistent with previous intervention and observational evidence. A systematic review and meta-analysis of undergraduate studies reported reductions in anxiety, depression, and stress following physical-activity interventions, although the certainty of evidence varied across outcomes (31). Prospective meta-analyses have also associated higher physical activity with lower subsequent risk of depression (34, 35), and a recent meta-analysis of 22 studies involving 66,683 university students found lower odds of depressive symptoms among more physically active students (36). Similar inverse associations have been reported among Chinese university students (39, 40).

Longitudinal evidence in university populations is less consistent. In a four-wave study, vigorous leisure-time physical activity was inversely associated with depressive symptoms at baseline, but changes in moderate or vigorous activity were not associated with corresponding changes in depressive symptoms (37). Another longitudinal study found that changes in vigorous and moderate leisure-time activity did not predict depressive-symptom trajectories, whereas increases in light activity were associated with slower symptom growth (38). These findings are compatible with the relatively small association observed in the present study and suggest that relationships between physical activity and student mental health may differ by activity intensity and over time. Greater psychological distress may also reduce students' motivation or willingness to engage in leisure-time activity.

The serial indirect association through chronic time pressure and leisure-time physical activity was statistically detectable but very small in magnitude (B = 0.010; standardized estimate = 0.009), particularly when compared with the indirect association through chronic time pressure alone (B = 0.182; standardized estimate = 0.180). Although the bootstrap confidence interval for H7 excluded zero, the size of the estimate remained modest, underscoring the importance of considering magnitude as well as statistical significance when interpreting indirect associations (55).

This result should therefore not be interpreted as evidence that leisure-time physical activity constitutes an important explanatory pathway linking perceived academic demands with psychological distress. Rather, within the present cross-sectional model, leisure-time physical activity contributed only a minimal additional concurrent association beyond chronic time pressure. The marked difference between H6 and H7 further suggests that most of the association between chronic time pressure and psychological distress was not captured by concurrent differences in leisure-time physical activity. Other responses to persistent time scarcity, including impaired recovery, sleep disruption, or repetitive cognitive engagement with academic tasks, may account for a larger share of this relationship and warrant direct examination in longitudinal research.

### 4.5 Public health and educational implications

The findings suggest that university mental-health promotion may benefit from attention to the organization of academic time in addition to individual-level psychological support. Because both perceived academic demands and chronic time pressure were associated with greater psychological distress, future institutional interventions could examine whether better coordination of major academic tasks and more predictable scheduling help reduce persistent time pressure among students. Such approaches would complement, rather than replace, counseling and other student-support services.

Physical-activity promotion may also need to account for perceived time constraints. Students reporting greater chronic time pressure also reported lower leisure-time physical activity, suggesting that limited discretionary time may reduce opportunities for participation. Flexible access to facilities, shorter activity options, and programs that can be integrated into fragmented schedules may therefore be useful targets for future evaluation. These strategies may be particularly relevant for students who perceive conventional exercise sessions as difficult to accommodate within academic routines.

The relatively small association between leisure-time physical activity and psychological distress, together with the small serial indirect association, indicates that physical activity represents only one part of the broader pattern observed in this study. Academic organization and physical-activity support should therefore be viewed as complementary rather than interchangeable approaches. Future intervention studies are needed to determine whether changes in academic scheduling, perceived time pressure, or access to activity opportunities lead to measurable improvements in student mental health.

### 4.6 Strengths, limitations, and future directions

Several features strengthen the study. Perceived academic demands, chronic time pressure, and psychological distress were modeled as distinct latent constructs, and the competing measurement models supported their empirical separation. The time-pressure item from the original academic-demand dimension was not included in the analyzed indicator set, reducing direct content overlap with chronic time pressure. Leisure-time physical activity was examined separately from work- and transport-related activity, consistent with the study's focus on discretionary time use. Reliability and convergent validity were satisfactory across the three latent constructs, and sensitivity analyses produced closely similar measurement and structural estimates.

The cross-sectional design limits conclusions about temporal ordering, and the indirect associations cannot establish longitudinal mediation (56). Perceived academic demands, chronic time pressure, and psychological distress were reported within the same questionnaire, leaving some potential for common method variance (53). Although Harman's single-factor test did not indicate a dominant single factor, this diagnostic has limited sensitivity and cannot rule out common method bias. Leisure-time physical activity was also self-reported and may therefore be affected by recall or reporting error despite previous psychometric evaluations of the GPAQ in international and Chinese university samples (45, 47).

The perceived-academic-demands measure used four of the five administered workload items, and TP2R showed a comparatively low standardized loading (0.394). TP2R was retained to preserve the original 13-item CTPI specification and avoid *post hoc* scale modification based solely on the present sample; sensitivity analyses excluding the item produced nearly identical measurement and structural estimates. Nevertheless, its low loading warrants further evaluation in university populations, including whether a shorter or re-specified CTPI could retain adequate construct coverage with improved measurement efficiency.

The sample was recruited through convenience sampling from 10 higher-education institutions in Jiangsu Province, which limits generalizability to other student populations. Jiangsu is an economically developed eastern coastal province, and the institutional resources, academic environments, and student circumstances represented in this sample may differ from those in inland or less economically developed regions of China. A conventional response rate could not be calculated because the number of students who received or viewed the survey invitation was not available. Institution- and class-level identifiers were not retained in the analytic dataset, so clustering across recruitment sites or classes could not be evaluated. If responses were positively correlated within institutions or classes, treating observations as independent may have underestimated standard errors and overstated the precision of some estimates. Residual confounding also remains possible because academic, behavioral, and psychosocial factors not included in the model may be related to both the focal predictors and psychological distress.

Future studies could use longitudinal or intensive repeated-measures designs to examine the temporal relationships among academic demands, perceived time pressure, leisure-time physical activity, and psychological distress. Broader probability-based samples, where feasible, would improve population representation, while objective or repeated measures of time use and physical activity could complement self-report data. Future multi-site studies should retain institution- and class-level identifiers, evaluate intraclass correlations, and, where warranted, account for hierarchical data structures using multilevel or cluster-adjusted methods. For prospective confirmatory studies, model-specific Monte Carlo procedures may be useful for sample-size and power planning (57).

## 5 Conclusion

Perceived academic demands and chronic time pressure were both associated with greater psychological distress among Chinese university students, while chronic time pressure was also associated with lower leisure-time physical activity. The indirect association through chronic time pressure was substantially larger than the serial indirect association additionally involving leisure-time physical activity, indicating that leisure-time physical activity made only a minimal additional contribution within the concurrent structural model. These findings highlight the relevance of chronic time pressure in the relationship between academic demands and psychological distress and suggest that student mental-health promotion may benefit from attention to both academic time organization and opportunities for physical activity. Because the study was cross-sectional, temporal ordering and causal direction cannot be established.

## Data Availability

The original contributions presented in the study are included in the article/Supplementary material, further inquiries can be directed to the corresponding authors.
