# Supplementary material for: Perceived academic demands and psychological distress among Chinese university students: structural associations involving chronic time pressure and leisure-time physical activity
Source: Front Public Health. 2026 Oct 5;14:1984531. doi: 10.3389/fpubh.2026.1984531 (PMC13677481; doi:10.3389/fpubh.2026.1984531)
Supplement: Supplementary file 1 [file Table_1.docx]

**Supplementary tables**

**Supplementary Table S1. Item wording, standardized factor loadings, squared multiple correlations, and source information for the latent-variable indicators**

| **Construct** | **Indicator** | **Item wording** | **Standardized loading** | **SMC** | **Source / adaptation** |
| --- | --- | --- | --- | --- | --- |
| Perceived academic demands | AD1 | Too many study tasks to complete. | 0.762 | 0.581 | Study Demands and Resources Scale, workload dimension (15); Chinese study wording |
| Perceived academic demands | AD3 | Completing study tasks requires exceptionally high effort. | 0.737 | 0.544 | Study Demands and Resources Scale, workload dimension (15); Chinese study wording |
| Perceived academic demands | AD4 | Study demands require sustained high attention. | 0.736 | 0.541 | Study Demands and Resources Scale, workload dimension (15); Chinese study wording |
| Perceived academic demands | AD5 | Study demands require remembering and processing substantial information. | 0.755 | 0.570 | Study Demands and Resources Scale, workload dimension (15); Chinese study wording |
| Chronic time pressure | TP1 | Often feel that there is not enough time in a day. | 0.795 | 0.632 | Chronic Time Pressure Inventory (19); Chinese study wording |
| Chronic time pressure | TP2R | Have enough time to do the things I want to do. | 0.394 | 0.155 | Chronic Time Pressure Inventory (19); Chinese study wording |
| Chronic time pressure | TP3 | Feel that everything must be fitted into limited time. | 0.773 | 0.598 | Chronic Time Pressure Inventory (19); Chinese study wording |
| Chronic time pressure | TP4 | Days pass quickly while things remain unfinished. | 0.773 | 0.598 | Chronic Time Pressure Inventory (19); Chinese study wording |
| Chronic time pressure | TP5 | Often feel rushed. | 0.771 | 0.594 | Chronic Time Pressure Inventory (19); Chinese study wording |
| Chronic time pressure | TP6R | Can control how time is arranged. ? | 0.713 | 0.508 | Chronic Time Pressure Inventory (19); Chinese study wording |
| Chronic time pressure | TP7 | Should have more free time to do preferred activities. | 0.501 | 0.251 | Chronic Time Pressure Inventory (19); Chinese study wording |
| Chronic time pressure | TP8 | Worry about whether time is being used effectively. | 0.742 | 0.551 | Chronic Time Pressure Inventory (19); Chinese study wording |
| Chronic time pressure | TP9R | Have enough time to prepare for things that need to be done. ? | 0.685 | 0.469 | Chronic Time Pressure Inventory (19); Chinese study wording |
| Chronic time pressure | TP10 | Often feel unable to complete planned tasks. | 0.744 | 0.553 | Chronic Time Pressure Inventory (19); Chinese study wording |
| Chronic time pressure | TP11 | Feel disappointed with how time is used. | 0.727 | 0.528 | Chronic Time Pressure Inventory (19); Chinese study wording |
| Chronic time pressure | TP12 | Always feel that there is not enough time. | 0.816 | 0.666 | Chronic Time Pressure Inventory (19); Chinese study wording |
| Chronic time pressure | TP13 | Often rush to complete necessary tasks. | 0.799 | 0.638 | Chronic Time Pressure Inventory (19); Chinese study wording |
| Psychological distress | PD1 | Felt nervous. | 0.767 | 0.589 | Kessler Psychological Distress Scale (K6) (48); Chinese undergraduate psychometric support (49) |
| Psychological distress | PD2 | Felt hopeless. | 0.796 | 0.633 | Kessler Psychological Distress Scale (K6) (48); Chinese undergraduate psychometric support (49) |
| Psychological distress | PD3 | Felt restless or fidgety. | 0.789 | 0.622 | Kessler Psychological Distress Scale (K6) (48); Chinese undergraduate psychometric support (49) |
| Psychological distress | PD4 | Felt so depressed that nothing could cheer them up. | 0.792 | 0.628 | Kessler Psychological Distress Scale (K6) (48); Chinese undergraduate psychometric support (49) |
| Psychological distress | PD5 | Felt that everything was an effort. | 0.749 | 0.561 | Kessler Psychological Distress Scale (K6) (48); Chinese undergraduate psychometric support (49) |
| Psychological distress | PD6 | Felt worthless. | 0.790 | 0.625 | Kessler Psychological Distress Scale (K6) (48); Chinese undergraduate psychometric support (49) |

Note. Standardized factor loadings and squared multiple correlations (SMCs) are from the hypothesized three-factor CFA in the final analytic sample (N = 1,390). SMC represents the proportion of variance in each observed indicator explained by its corresponding latent factor. TP2, TP6, and TP9 were reverse scored before CFA and SEM estimation; their original item wording is shown in the table. AD2 was administered as part of the original workload dimension but was excluded from the analyzed perceived-academic-demands indicator set because it explicitly referred to studying or completing tasks under time pressure. Accordingly, AD2 is not included in this table. Item wording shown in English is a reporting translation of the Chinese-administered questionnaire wording. Leisure-time physical activity was modeled as an observed variable and was therefore not included in the CFA. PAD, perceived academic demands; TP, chronic time pressure; PD, psychological distress.

**Supplementary Table S2. Covariate associations with endogenous variables in the adjusted structural model**

| **Covariate** | **Outcome** | **B** | **SE** | **β** | **p** |
| --- | --- | --- | --- | --- | --- |
| Age | Chronic time pressure | −0.003 | 0.018 | −0.004 | 0.871 |
| Women vs men | Chronic time pressure | 0.036 | 0.047 | 0.019 | 0.440 |
| Prefer not to answer vs men | Chronic time pressure | 0.105 | 0.170 | 0.015 | 0.535 |
| BMI | Chronic time pressure | −0.007 | 0.009 | −0.018 | 0.455 |
| Weekly organized physical activity | Chronic time pressure | −0.024 | 0.019 | −0.031 | 0.204 |
| Family financial pressure | Chronic time pressure | 0.027 | 0.020 | 0.032 | 0.186 |
| Another gender identity vs men | Chronic time pressure | 0.240 | 0.196 | 0.030 | 0.221 |
| Age | Leisure-time physical activity | 0.028 | 0.062 | 0.012 | 0.647 |
| Women vs men | Leisure-time physical activity | 0.176 | 0.159 | 0.029 | 0.270 |
| Prefer not to answer vs men | Leisure-time physical activity | −0.250 | 0.572 | −0.011 | 0.663 |
| BMI | Leisure-time physical activity | 0.027 | 0.031 | 0.023 | 0.388 |
| Weekly organized physical activity | Leisure-time physical activity | 0.175 | 0.064 | 0.072 | 0.006 |
| Family financial pressure | Leisure-time physical activity | 0.038 | 0.069 | 0.014 | 0.585 |
| Another gender identity vs men | Leisure-time physical activity | −1.846 | 0.662 | −0.073 | 0.005 |
| Age | Psychological distress | 0.020 | 0.016 | 0.029 | 0.200 |
| Women vs men | Psychological distress | 0.008 | 0.041 | 0.004 | 0.852 |
| Prefer not to answer vs men | Psychological distress | 0.064 | 0.147 | 0.010 | 0.663 |
| BMI | Psychological distress | −0.006 | 0.008 | −0.018 | 0.431 |
| Weekly organized physical activity | Psychological distress | 0.031 | 0.016 | 0.043 | 0.058 |
| Family financial pressure | Psychological distress | 0.052 | 0.018 | 0.066 | 0.004 |
| Another gender identity vs men | Psychological distress | −0.075 | 0.171 | −0.010 | 0.662 |

Note: Men were the reference category for the three gender dummy variables. B, unstandardized coefficient; SE, standard error; β, standardized coefficient. Weekly organized physical activity refers to weekly hours of physical education or other university-organized physical activity.

**Supplementary Table S3. Sensitivity analysis excluding TP2R from the Chronic Time Pressure Inventory**

| **Parameter** | **Primary model (13-item CTPI)** | **Sensitivity model (TP2R excluded)** |
| --- | --- | --- |
| **Measurement model (CFA)** |  |  |
| χ² (df) | 816.39 (227) | 769.50 (206) |
| χ²/df | 3.596 | 3.735 |
| CFI | 0.967 | 0.968 |
| TLI | 0.964 | 0.965 |
| RMSEA (90% CI) | 0.043 [0.040, 0.046] | 0.044 [0.041, 0.048] |
| SRMR | 0.0276 | 0.0277 |
| **Adjusted structural model** |  |  |
| χ² (df) | 1006.51 (387) | 944.51 (358) |
| χ²/df | 2.601 | 2.638 |
| CFI | 0.966 | 0.968 |
| TLI | 0.959 | 0.961 |
| RMSEA (90% CI) | 0.034 [0.031, 0.037] | 0.034 [0.032, 0.037] |
| SRMR | 0.0235 | 0.0230 |
| R² chronic time pressure | 0.299 | 0.299 |
| R² leisure-time physical activity | 0.063 | 0.063 |
| R² psychological distress | 0.437 | 0.437 |
| Perceived academic demands → chronic time pressure, β | 0.539 | 0.539 |
| Chronic time pressure → leisure-time physical activity, β | −0.179 | −0.180 |
| Perceived academic demands → psychological distress, β | 0.382 | 0.383 |
| Chronic time pressure → psychological distress, β | 0.334 | 0.333 |
| Leisure-time physical activity → psychological distress, β | −0.097 | −0.097 |
| Perceived academic demands → leisure-time physical activity, β | −0.058 | −0.057 |
| H6, B [95% BC CI] | 0.182 [0.134, 0.236] | 0.182 [0.133, 0.236] |
| H7, B [95% BC CI] | 0.010 [0.005, 0.018] | 0.010 [0.005, 0.018] |

Note: The sensitivity model excluded TP2R only; all other measurement and structural specifications were unchanged. BC CI, bias-corrected bootstrap confidence interval; bootstrap resamples = 5,000 for H6 and H7. SRMR values were obtained using the same Amos Standardized RMR plugin workflow.

**Supplementary Table S4. Correlations of self-reported academic time-use indicators with perceived academic demands and chronic time pressure**

| **Academic time-use indicator** | **Perceived academic demands** | **Chronic time pressure** |
| --- | --- | --- |
| Formal class hours/week | −0.012 (p = 0.646) | −0.011 (p = 0.693) |
| Extracurricular study hours/week | 0.556 (p < 0.001) | 0.388 (p < 0.001) |

Note: Values are Pearson correlation coefficients with two-tailed p values in parentheses. N = 1,390. Formal class hours and extracurricular study hours were self-reported.
