# Supplementary material for: Perceived academic demands and psychological distress among Chinese university students: structural associations involving chronic time pressure and leisure-time physical activity
Source: Front Public Health. 2026 Oct 5;14:1984531. doi: 10.3389/fpubh.2026.1984531 (PMC13677481; doi:10.3389/fpubh.2026.1984531)
Supplement: Supplementary file 2 [file Table_2.docx]

**大学生学业要求、时间压力、体力活动与心理健康调查问卷**

# 调查说明与电子知情同意

您好！本调查旨在了解大学生的学习任务、时间感受、日常体力活动和心理健康状况。问卷没有正确或错误答案，请根据您的真实情况作答。

问卷内容仅用于学术研究，所有数据将匿名处理。您可以随时停止作答，不会受到任何不利影响。

点击“同意并继续”表示您已经阅读上述说明，自愿参与本研究。

□ 同意参加	□ 不同意参加

# 第一部分 筛查及基本信息

**A1. 您目前是否为全日制本科生？**

□ 是	□ 否

**A2. 您的年龄**

_____ 周岁

**A3. 您的性别**

□ 男	□ 女	□ 其他	□ 不愿回答

**A4. 您目前所在年级**

□ 大一	□ 大二	□ 大三	□ 大四	□ 五年制专业大五

**A5. 您的身高**

_____ 厘米

**A6. 您的体重**

_____ 千克

**A7. 本学期您平均每周参加多少小时的体育课或学校统一安排的体育活动？**

_____ 小时/周

**A8. 过去 4 周，您平均每周有多少小时的正式课程？包括线下课、线上同步课和实验课。**

_____ 小时/周

**A9. 过去 4 周，您平均每周有多少小时用于课外学习？包括完成作业、复习、阅读、实验准备、课程项目和备考，不包括正式上课时间。**

_____ 小时/周

**A10. 过去 4 周，您平均每天实际睡眠时间为：**

_____ 小时/天

**A11. 您目前是否感受到明显的家庭经济压力？**

1 完全没有	2 较少	3 一般	4 较大	5 非常大

# 第二部分 感知学业要求

请根据过去 4 周的学习情况，选择最符合您的答案。

计分方式：1＝从不；2＝很少；3＝有时；4＝经常；5＝总是。

**AD1 我需要完成的学习任务过多。**

1	2	3	4	5

**AD2 我常常需要在时间压力下学习或完成任务。**

1	2	3	4	5

**AD3 为了完成学习任务，我不得不付出格外大的努力。**

1	2	3	4	5

**AD4 我的学习要求我持续保持高度注意。**

1	2	3	4	5

**AD5 我的学习要求我同时记住和处理许多信息。**

1	2	3	4	5

# 第三部分 慢性时间压力

请根据过去 4 周的通常感受，判断您对以下陈述的同意程度。

计分方式：1＝非常不同意；2＝不同意；3＝不确定；4＝同意；5＝非常同意。

**TP1 我常觉得一天的时间根本不够用。**

1	2	3	4	5

**TP2 我有足够的时间做自己想做的事情。**

1	2	3	4	5

**TP3 我感到必须把所有事情都安排进有限的时间里。**

1	2	3	4	5

**TP4 日子过得很快，而我总有事情没有完成。**

1	2	3	4	5

**TP5 我经常处于匆忙状态。**

1	2	3	4	5

**TP6 我能够掌控自己如何安排时间。**

1	2	3	4	5

**TP7 我应该拥有更多自由时间去做自己喜欢的事情。**

1	2	3	4	5

**TP8 我会担心自己是否把时间使用得足够合理。**

1	2	3	4	5

**TP9 我有足够的时间为要做的事情做好准备。**

1	2	3	4	5

**TP10 我常觉得无法完成原本计划完成的事情。**

1	2	3	4	5

**TP11 我对自己使用时间的方式感到失望。**

1	2	3	4	5

**TP12 我总是觉得时间不够。**

1	2	3	4	5

**TP13 我常常匆忙地完成必须做的事情。**

1	2	3	4	5

# 第四部分 身体活动

请根据您通常一周的情况回答以下问题。

**PA1 学习、兼职或日常事务中的高强度身体活动**

在学习、兼职工作或其他日常事务中，您是否进行过需要较大体力、使呼吸或心率显著加快的高强度身体活动？

是否参加：□ 是	□ 否

每周天数：_____ 天/周

每天持续时间：_____ 分钟/天

**PA2 学习、兼职或日常事务中的中等强度身体活动**

在学习、兼职工作或其他日常事务中，您是否进行过需要中等体力、使呼吸或心率适度加快的中等强度身体活动？

是否参加：□ 是	□ 否

每周天数：_____ 天/周

每天持续时间：_____ 分钟/天

**PA3 步行或骑自行车交通活动**

在通常一周中，您是否步行或骑自行车往返不同地点，例如上课、兼职、购物或其他日常出行？

是否参加：□ 是	□ 否

每周天数：_____ 天/周

每天持续时间：_____ 分钟/天

**PA4 休闲时间高强度身体活动**

包括跑步、快速游泳、竞技球类、快速骑行或其他使呼吸和心率显著增加的休闲运动。

是否参加：□ 是	□ 否

每周天数：_____ 天/周

每天持续时间：_____ 分钟/天

**PA5 休闲时间中等强度身体活动**

包括快走、一般速度骑行、休闲游泳、健身操或其他使呼吸和心率适度增加的休闲运动。

是否参加：□ 是	□ 否

每周天数：_____ 天/周

每天持续时间：_____ 分钟/天

**PA6 久坐时间**

典型一天坐着或斜躺的总时间：_____ 分钟/天

# 第五部分 心理困扰

下面的问题询问您在过去 30 天中的感受。请选择您有多长时间出现以下感受。

计分方式：0＝从来没有；1＝少数时间；2＝有些时间；3＝大部分时间；4＝所有时间。

**PD1 您有多长时间感到紧张？**

0	1	2	3	4

**PD2 您有多长时间感到绝望？**

0	1	2	3	4

**PD3 您有多长时间感到坐立不安或烦躁不安？**

0	1	2	3	4

**PD4 您有多长时间感到情绪非常低落，以至于没有什么事情能使您振作起来？**

0	1	2	3	4

**PD5 您有多长时间感到做任何事情都很费劲？**

0	1	2	3	4

**PD6 您有多长时间感到自己没有价值？**

0	1	2	3	4

感谢您的参与！
