# Supplementary material for: Perceived academic demands and psychological distress among Chinese university students: structural associations involving chronic time pressure and leisure-time physical activity
Source: Front Public Health. 2026 Oct 5;14:1984531. doi: 10.3389/fpubh.2026.1984531 (PMC13677481; doi:10.3389/fpubh.2026.1984531)
Supplement: Supplementary file 3 [file Table_3.docx]

**Questionnaire on Academic Demands, Time Pressure, Leisure-Time Physical Activity, and Mental Health among University Students**

# Survey Information and Electronic Informed Consent

Hello. This survey aims to understand university students' academic tasks, perceptions of time, daily physical activity, and mental health. There are no right or wrong answers. Please respond according to your actual situation.

The questionnaire is used solely for academic research, and all data will be anonymized. You may stop participating at any time without any adverse consequences.

By selecting "Agree and continue," you indicate that you have read the information above and voluntarily agree to participate in this study.

□ Agree to participate	□ Do not agree to participate

# Part I. Screening and Basic Information

**A1. Are you currently a full-time undergraduate student?**

□ Yes	□ No

**A2. Your age**

_____ years

**A3. Your gender**

□ Male	□ Female	□ Other	□ Prefer not to answer

**A4. Your current year of study**

□ First year	□ Second year	□ Third year	□ Fourth year	□ Fifth year in a five-year program

**A5. Your height**

_____ cm

**A6. Your weight**

_____ kg

**A7. During the current semester, how many hours per week, on average, do you participate in physical education classes or other university-organized physical activities?**

_____ hours/week

**A8. During the past 4 weeks, how many hours per week, on average, have you spent in formal classes? Include in-person classes, synchronous online classes, and laboratory classes.**

_____ hours/week

**A9. During the past 4 weeks, how many hours per week, on average, have you spent on extracurricular study? Include assignments, review, reading, laboratory preparation, course projects, and examination preparation, but exclude formal class time.**

_____ hours/week

**A10. During the past 4 weeks, what was your average actual sleep duration per day?**

_____ hours/day

**A11. Do you currently experience substantial family financial pressure?**

1 None at all	2 Low	3 Moderate	4 High	5 Very high

# Part II. Perceived Academic Demands

Please select the response that best describes your study experience during the past 4 weeks.

Response scale: 1 = Never; 2 = Rarely; 3 = Sometimes; 4 = Often; 5 = Always.

**AD1. I have too many study tasks to complete.**

1	2	3	4	5

**AD2****. I often have to study or complete tasks under time pressure.**

1	2	3	4	5

**AD3. I have to make an exceptionally large effort to complete my study tasks.**

1	2	3	4	5

**AD4. My studies require me to maintain a high level of attention continuously.**

1	2	3	4	5

**AD5. My studies require me to remember and process a large amount of information at the same time.**

1	2	3	4	5

# Part III. Chronic Time Pressure

Based on how you have generally felt during the past 4 weeks, indicate how much you agree with each statement.

Response scale: 1 = Strongly disagree; 2 = Disagree; 3 = Neither agree nor disagree; 4 = Agree; 5 = Strongly agree.

**TP1. I often feel that there is simply not enough time in the day.**

1	2	3	4	5

**TP2. I have enough time to do the things I want to do.**

1	2	3	4	5

**TP3. I feel that I have to fit everything into a limited amount of time.**

1	2	3	4	5

**TP4. The days pass quickly, and I always have things left unfinished.**

1	2	3	4	5

**TP5. I am often in a hurry.**

1	2	3	4	5

**TP6. I am able to control how I organize my time.**

1	2	3	4	5

**TP7. I should have more free time to do the things I enjoy.**

1	2	3	4	5

**TP8. I worry about whether I am using my time well enough.**

1	2	3	4	5

**TP9. I have enough time to prepare properly for the things I need to do.**

1	2	3	4	5

**TP10. I often feel unable to complete the things I originally planned to finish.**

1	2	3	4	5

**TP11. I feel disappointed with the way I use my time.**

1	2	3	4	5

**TP12. I always feel that I do not have enough time.**

1	2	3	4	5

**TP13. I often rush to finish the things that I have to do.**

1	2	3	4	5

# Part IV. Leisure-Time Physical Activity and Sedentary Time

The following items were used in this study to assess leisure-time physical activity and sedentary time.

**PA4. Vigorous-intensity leisure-time physical activity**

Examples include running, fast swimming, competitive ball sports, fast cycling, or other leisure activities that substantially increase breathing and heart rate.

Do you participate? □ Yes	□ No

Days per week: _____ days/week

Duration per day: _____ minutes/day

**PA5. Moderate-intensity leisure-time physical activity**

Examples include brisk walking, cycling at a normal pace, recreational swimming, aerobics, or other leisure activities that moderately increase breathing and heart rate.

Do you participate? □ Yes	□ No

Days per week: _____ days/week

Duration per day: _____ minutes/day

**PA6. Sedentary time**

Total time spent sitting or reclining on a typical day: _____ minutes/day

# Part V. Psychological Distress

The following questions ask about how you felt during the past 30 days. Please indicate how often you experienced each feeling.

Response scale: 0 = None of the time; 1 = A little of the time; 2 = Some of the time; 3 = Most of the time; 4 = All of the time.

**PD1. How often did you feel nervous?**

0	1	2	3	4

**PD2. How often did you feel hopeless?**

0	1	2	3	4

**PD3. How often did you feel restless or fidgety?**

0	1	2	3	4

**PD4. How often did you feel so depressed that nothing could cheer you up?**

0	1	2	3	4

**PD5. How often did you feel that everything was an effort?**

0	1	2	3	4

**PD6. How often did you feel worthless?**

0	1	2	3	4

Thank you for your participation.
